# Covaxin-Induced Lymphocytic Myocarditis

**DOI:** 10.7759/cureus.26759

**Published:** 2022-07-11

**Authors:** Nitish Mittal, Dushyant Pawar, Kanak Parmar, Zhaunn Sly, Gaspar Del Rio-Pertuz, Mohammad M Ansari, Nandini Nair

**Affiliations:** 1 Internal Medicine, University of Texas Health Sciences Center, Houston, USA; 2 Internal Medicine, Texas Tech University Health Sciences Center, Lubbock, USA; 3 Cardiology, Texas Tech University Health Sciences Center, Lubbock, USA

**Keywords:** covid-19 vaccination myocarditis, heart failure, endomyocardial biopsy, vaccine, covid-19

## Abstract

We report the case of a young adult male with endomyocardial biopsy-proven lymphocytic myocarditis following Covaxin administration. Covaxin differs from the mRNA vaccines in that it is an inactivated virus developed using the whole virion inactivated using the Vero cell platform. We successfully managed the patient with complete resolution of symptoms.

## Introduction

COVID-19 is a global pandemic that has brought new challenges to our health system. The different types of syndromes of acute respiratory syndrome coronavirus 2 (SARS-CoV-2) vaccines have shown to significantly reduce the risk of contracting COVID-19 disease. However, despite safety and efficacy, there are some side effects. Recently, the safety committee of the US Centers for Disease Control and Prevention stated that there was a likely association between SARS-CoV-2 vaccines and myocarditis/pericarditis. Cases of Covaxin-associated myocarditis have rarely been reported in the literature. We report the case of a young adult male with endomyocardial biopsy (EMB) proven lymphocytic myocarditis following Covaxin administration.

## Case presentation

**Figure 1:**
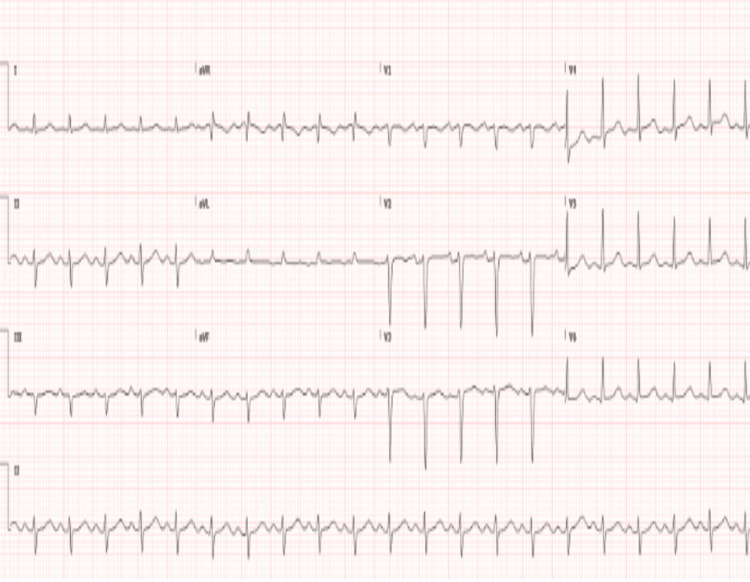
EKG showing tachycardia with left axis deviation

**Figure 2:**
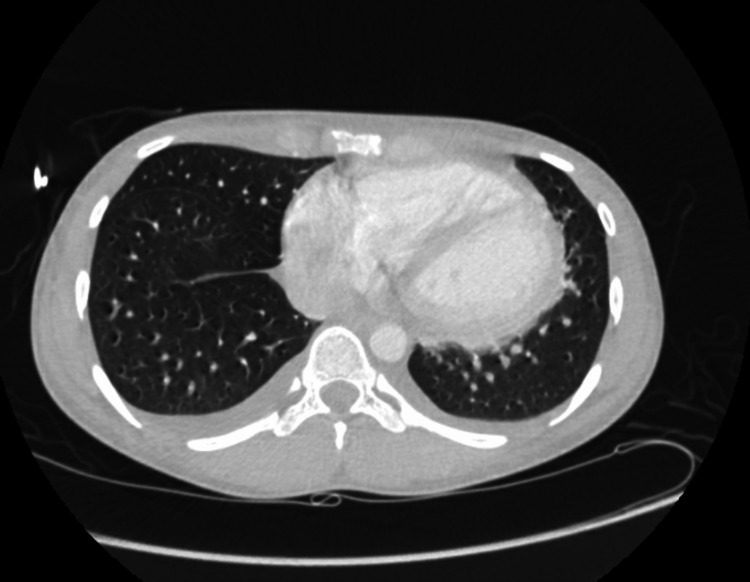
CT chest (transverse) showing cardiomegaly and some signs of fluid overload

**Figure 3:**
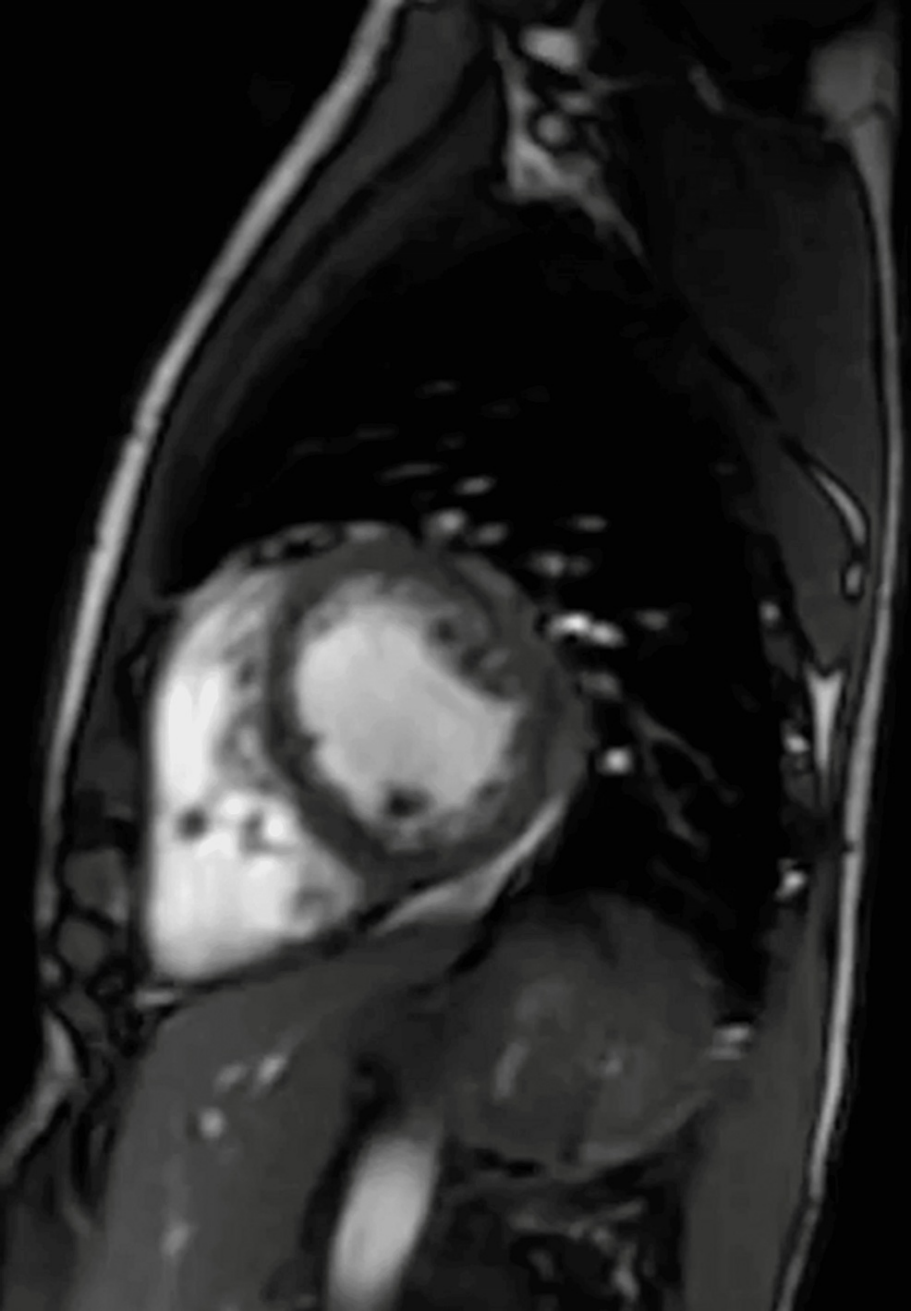
Cardiac MRI short axis view without abnormal late gadolinium enhancement or abnormal signal to suggest myocarditis or infiltrative cardiomyopathy

**Figure 4:**
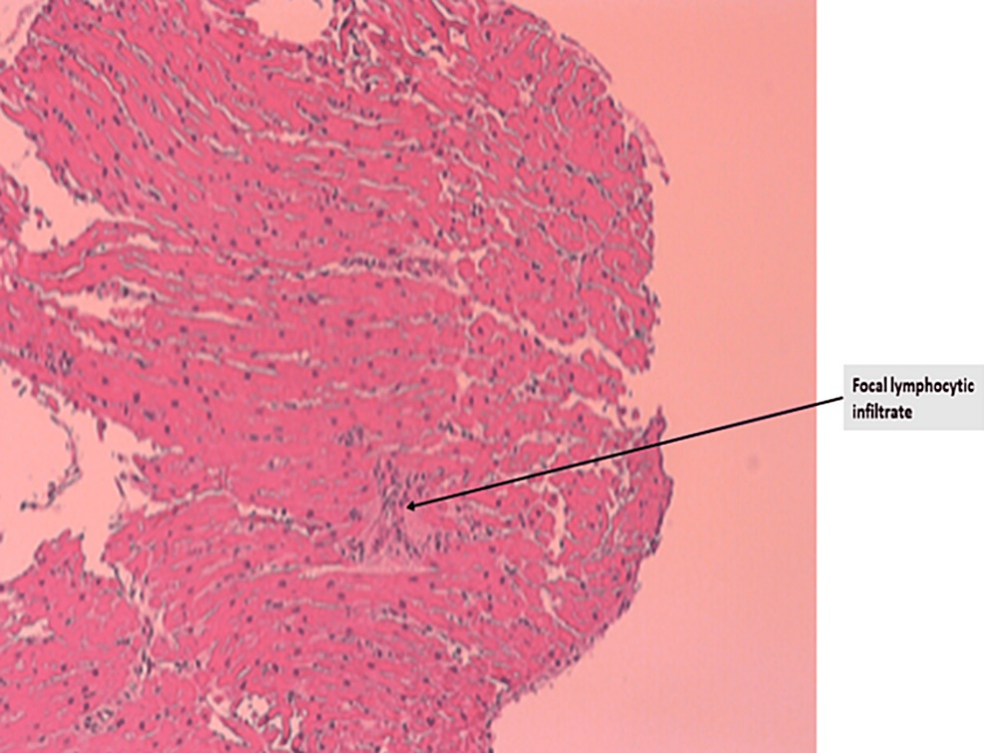
Endomyocardial biopsy showing lymphocytic myocarditis

A South Asian male in his early 20s presented to a local hospital in the United States with worsening lower extremity swelling, weakness, and pain. Patient had arrived on a long-distance flight a month ago and reported symptoms beginning a week prior to traveling but had worsened after arrival to the United States. The pain was described as a deep crampy pain with left side worse than the right. Approximately 14 days prior to presentation, he had begun to develop significant cramping and weakness in his lower extremities limiting his ability to ambulate. He denied any such episodes in the past or any sick contacts with fever, chills, dyspnea, chest pain, nausea, vomiting, or dysuria. He reported upper respiratory symptoms a few months prior to this event but was never tested for any infection. He had received the first dose of Covaxin two months prior to presentation, which was the only incident of significance between the occurrence of upper respiratory symptoms and onset of bilateral leg swelling and pain. He reported no immediate symptoms following vaccination. He is an occasional social drinker but uses no tobacco or recreational drugs. Upon presentation, he was afebrile, normotensive, tachycardic (124 beats/min), and oxygen saturation at 99% on room air. The physical exam revealed bilateral lower extremities edema, intact pulses, bibasilar crackles, and diminished heart sounds. Laboratory values are described in Table 1. The patient was healthy with no past medical history with no significant family history. Our differential list was broad including viral and vaccine-associated myocarditis, myositis, toxins, pulmonary embolism, autoimmune disease, and systemic disorders. The assessment of the 12-lead electrocardiogram (EKG) showed tachycardia with left axis deviation (Figure 1). The computed tomography (CT) chest scan showed cardiomegaly and signs of fluid overload (Figure 2). The transthoracic echo (TTE) showed a 40%-44% ejection fraction (EF) with mild global hypokinesis of all myocardial segments of the left ventricle. The following day, cardiac magnetic resonance imaging was performed showing small pericardial effusion (Figure 3). The LVEF was 49% and the RVEF was 39%. No other abnormalities were noted. Bilateral lower extremity Doppler showed no evidence of deep or superficial venous thrombosis. After stabilizing the vitals, right heart catheterization was performed with EMB, which showed active lymphocytic myocarditis with infiltrates and focal myocyte injury (Figure 4).

**Table 1:** Laboratory results

Laboratory	Results	Normal range
Lactate	7.7 mmol/L	0.5-2.2 mmol/L
Pro-Brain Natriuretic Peptide (BNP)	3,437 pg/mL	<124 pg/mL
Troponin T High Sensitivity	47.1 ng/L	<19 ng/L
Creatine kinase	691 intl units/L	26-308 intl units/L
Lactate dehydrogenase	417 units/L	135-225 units/L
D-Dimer	939 ng/ml FEU	<500 ng/ml FEU
Coxsackie A IgM	Negative	
Coxsackie B IgM	Negative	
Coxsackie A IgG	Negative	
Coxsackie B IgG	Negative	
Influenza A IgM	Negative	
Influenza B IgM	Negative	
Influenza A IgG	Negative	
Influenza B IgG	Negative	

Autoimmune workup was negative along with negative infectious disease screen including hepatitis panel, Epstein Barr virus, human immunodeficiency virus, tuberculosis, syphilis, and herpes simplex virus. The patient was started on diuresis with furosemide, and heart rate was controlled with metoprolol succinate. Furosemide dose was up titrated with a near-complete resolution of edema. The myocarditis was treated with a tapering dose of methylprednisolone for seven days. The symptoms of the patient improved with down-trending lab values and the patient was discharged on metoprolol/furosemide/methylprednisolone taper. Follow-up TTE as an outpatient three months later showed normalization of EF of 60% with preserved RV function.

## Discussion

Lymphocytic myocarditis following Covaxin administration has not been reported before. Emergency use authorization for Covaxin to prevent COVID-19 caused by SARS-CoV-2 was developed by Bharat Biotech, an Indian drug company in collaboration with the National Institute of Virology, Pune, India, and the Indian Council of Medical Research [1]. This vaccination series comprises two doses given four weeks apart. Covaxin differs from the mRNA vaccines in that it is an inactivated virus vaccine developed using the whole virion inactivated using the Vero cell platform [2]. The Vero cell platform was utilized to accelerate the process development [2]. Our case is unique in shedding light on post-vaccination myocarditis in a young adult male without any other medical issues with an inactivated virus vaccine. It is important to emphasize that we were not able to completely exclude that our patient had prior silent COVID-19 infection as he never got tested, which may have increased his risk for vaccine-associated myocarditis. In addition, the side effects reported in the phase 1 trial and three-month follow-up of Covaxin were essentially injection site pain, headache, fatigue, fever, nausea, or vomiting [3,4].

Myocarditis was definitively diagnosed on EMB [5]. Viral myocarditis is the most common form of lymphocytic myocarditis [6]. A case report of hyper-eosinophilic syndrome with myocarditis in a healthy young adult male following Covaxin vaccination has been described previously [7]. Similarly, prior reports have linked myocarditis to different vaccinations, including smallpox and influenza [8,9]. The underlying etiology is thought to be provoked by an autoimmune response in the setting of molecular mimicry versus a nonspecific inflammatory process [10,11]. The various proposed mechanisms for myocarditis include immune response to mRNA; activation of immunologic pathways eventually resulting in the systemic immune response that may be exaggerated in predisposed individuals resulting in organ damage; molecular mimicry between the SARS-CoV-2 spike protein and self-antigens on myocardium; delayed hypersensitivity by re-exposure to a spike protein antigen, with the first exposure being either prior infection or 1st vaccine dose; eosinophilic infiltration of cardiac tissue causing myocardial injury [12]. Interestingly, male predominance of COVID-19 mRNA vaccine-related myocarditis has been reported, which could possibly be due to underdiagnosis in women or the effect of testosterone in the inhibition of the anti-inflammatory cells [12,13]. However, data on Covaxin-induced myocarditis remains limited.

The management of vaccine-induced myocarditis is mainly supportive. It includes steroids, non-steroidal anti-inflammatory drugs, intravenous immunoglobulin, and guideline-directed medical therapy for heart failure. Data from case reports in the literature suggest that COVID-19 vaccine-related myocarditis clinical symptoms resolve typically within a week with preservation/restoration of cardiac function [14]. Our patient showed improvement after initiating methylprednisone, and the clinical symptoms subsided significantly within a week. Hence, Covaxin induced lymphocytic myocarditis should be included in the differential diagnosis.

## Conclusions

Covaxin-induced lymphocytic myocarditis has not been reported before. The management is mainly supportive including steroids, intravenous immunoglobulin, pain medications, and guideline-directed medical therapy for heart failure. Despite the side effects associated with the vaccine, the benefit of vaccination greatly outweighs the risks. Myocarditis, though rare with Covaxin, should be on the differential list for any COVID-19 vaccine-related cardiac symptoms to ensure early diagnosis and prompt management.
